# Environmental Conditions of Dance Rooms and Its Impact on Dance Conservatories Teachers’ Health (An Andalusian Study)

**DOI:** 10.3390/ijerph18105319

**Published:** 2021-05-17

**Authors:** María Dolores Redel-Macías, Carmen del Rio, Pedro Arezes, Pilar Aparicio-Martínez, Antonio José Cubero-Atienza

**Affiliations:** 1 Department Rural Engineering, EPS, Edificio Leonardo da Vinci, Campus de Rabanales, Universidad de Córdoba, 14071 Córdoba, Spain; ir1cuata@uco.es; 2 High Professional Dance Conservatory “Luis del Rio”, 14003 Córdoba, Spain; mcbolera@hotmail.com; 3 ALGORITMI Centre, School of Engineering, University of Minho, 4704-553 Guimaraes, Portugal; pres-pedro.arezes@eng.uminho.pt; 4 Department of Nursing, Pharmacology and Physiotherapy, Faculty of Medicine and Nursing, Universidad de Córdoba, 14071 Córdoba, Spain; n32apmap@uco.es

**Keywords:** dance conservatory, noise exposure, thermal conditions, health problems, occupational safety and health

## Abstract

Dance teachers have to be in long hours dancing. That entails repetitive movements, loud live music, and as well as forcing their voices. These demands can implicate severe health problems and other kind of illness as discomfort, stress, etc. However, the Spanish Ministry of Health only recognize as professional disease for this line of work, the vocal nodules. For this reason, this research studies the health problems in dance teachers in Andalusia, correlating the results of a survey carried out in different conservatories from Andalusia with measurement of noise emissions levels, assessment of noise exposure, and assessment of thermal environment in the classes measuring the thermal environment variables. To the authors’ knowledge, this is the first study where the influence of several sounds, such as tapping, castanets, and live music, on the health of dance teachers, musicians, and singers during flamenco classes has been researched. Results showed a correlation between some diseases, such as stress and the high level of sound in the classes. The sound levels were well above those established by European regulations reaching values higher than 85 dB(A) as equivalent continuous sound levels during the class time. This European regulation is stablished for an 8 h/day period, five days per week. The thermal environments are no adequate for this activity, mainly for high temperatures in Cordoba during summer. To improve the current working conditions, some recommendations were given to reduce the number of class hours and establish rest shifts, provide more information on health risks, or renovate the floor of some classrooms.

## 1. Introduction

Dance is a health-related exercise or fitness that improves cardiovascular parameters, oxygen consumption, muscular power, strength, and endurance, as well as decreases fat-free body mass and improves flexibility and elasticity [1,2]. Nevertheless, the competitive dance also causes injuries and muscular and bone damages due to the segments in repetitive rhythmic movements [3,4]. These repetitive rhythmic movements are the leading risk factor that contributes to the injuries [5,6]. Besides, the environmental conditions also play a relevant role. One study that focused on Australian dancers indicated how part-time dancers, usually choreographers or teachers, had a higher prevalence of injuries and health problems, which could be linked to the different scenarios or changes of the working environment [7]. Despite the prevalence of health problems in choreographers or teachers, most studies focused on the students or did not include the environmental conditions [8].

For dance, specific environmental conditions are required, such as adequate music, spaces, and thermal conditions [9]. Nonetheless, loud music and thermal conditions have side effects on health, from hearing disabilities to vascular dysfunction [10,11,12], being more commonly studied on conservatory musicians and focused on students [11,13]. These health risks are related to parasympathetic stimulation and vibrations [11,13].

One specific dance that originates high vibrations exposure is flamenco. Flamenco, a Spanish folk dance, is accompanied by tapping of the feet and several musical instruments; therefore, there is need for at least three people (the dance teacher, the singer and clapper, and the guitarist) so that the dancing students can have their lesson [14]. In most Spanish dancing schools, the music can be live or through Wi-Fi systems amplifying the vibrations and rhythmic patterns. Additionally to the music created, the tapping of the heels and castanets sound is integrated into the tempo, so specific modifications and musical sequences to carry out specific dance and musical practices are realized [14]. There are over 100 conservatory dance professionals in Spain, being the main worry (of this study), the health of these professionals regarding the exposure to hazardous working conditions such as noise, vibrations, and environmental conditions. In particular, the noise generated inside the classrooms, the vibrations transmitted and felt by their bodies [15], and the possible implications derived from this exposure [16], as well as the exposure to environmental conditions (mainly thermal conditions) that make their work harder. Among the noise implications, the most frequent are the hearing problems and voice-related problems due to the lack of intelligibility, that make necessary shouting during classes [16]. Regarding the vibrations, anatomic and functional problems are caused by the absorbing impacts from the percussion of the instruments used in repetitive dancing (zapateado or foot/heel tapping, castanets, among others) [12]. On the other hand, the rooms’ incorrect soundproofing leads to poor working conditions [17]. The thermal environment in classrooms is another important factor of health, as mentioned before, mainly in the summer.

Due to the existence of these problems in the conservatories and to the negative implications of those problems for the groups of affected workers, the Territorial Department for Education, Culture, and Sport of the Regional Government of Andalusia published an Information Handbook on Work-Related Risks aimed at the teachers [18]. This guide includes specific related issues, hygienic work-related risks within a classroom, such as exposure to noise and biological agents, ergonomic risks such as high noise levels during classes that make impossible an adequate intelligibility, and forced body postures, and psychosocial risks such as stress. 

**Figure 1:**
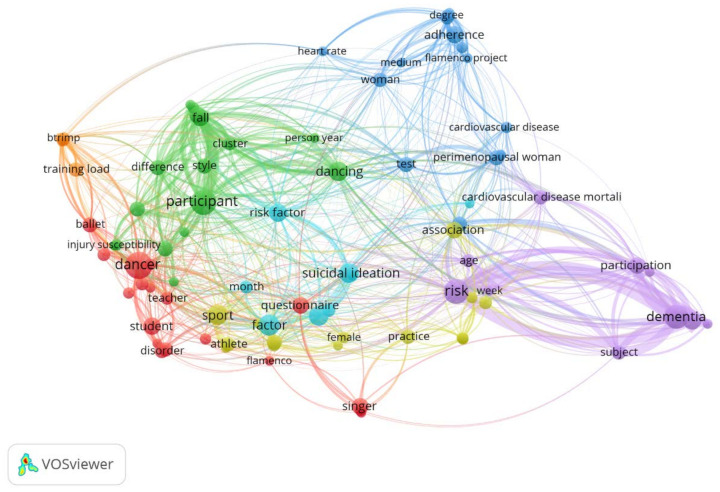
The main topics researched in dance education.

No specific previous references including these study elements in a conservatory of dance have been found. However, there are some isolated works on flamenco, mainly from the point of view of the activity and the physical preparation as regards heel tapping [19,20,21] or from the viewpoint of injuries related to the positions of the players’ hands/wrists when playing the piano and the guitar [22]. Based on that, a literature review was carried out to determine the current literature regarding dance teachers’ health. The keywords used for this research in Web of Science, ScienceDirect, Scopus, and PubMed were “dance teacher” and “risk prevention”. Although the search included all years until 2020, the latest records found were dated 2014. The most recent results are those related to “risk factors”, “injury”, and “prevention”. The results (Figure 1) indicated a limited number of studies compared to other research areas, such as Ergonomics [23].

A reasonable number of studies on teachers’ voice disorder for general education are related to the noise level in the classes or the effect of the classroom acoustics [24,25]. In fact, in the United Kingdom (UK), the document BB93 Acoustic Design of Schools gives design guidance both for refurbished and the new classrooms since, according to the survey carried out by London South Bank University, over 60% of the teachers surveyed had experienced voice problems during their career [24,26,27]. However, only a few researches about the exposure to noise among dance teachers have been found [28].

A recent study [19] has shown the extent and characteristics of injuries in contemporary dance students. One hundred thirty-four students of Bachelor dance from Codarts University of Arts (Rotterdam, the Netherlands) were evaluated during one academic year. Results showed that 97% of students had at least one injury, mental complaint, or other health problem. The authors checked the monthly injury proportion, and finally, they concluded that the injury incidence rate per 1000 h of dance exposure was 1.9 (95% CI 1.7 to 2.2). Moreover, they were able to establish that 30% were ankle/foot injuries, 17% were lower back injuries, and 15% were knee injuries. Another study highlighted the importance of an adequate dance floor to prevent injuries [20]. A total of 86 institutions among theatres and education establishments were surveyed in Germany. The conclusions were that inappropriate floors could be significantly responsible for chronic injuries and overuse complaints. Van Seters et al. [21] determined if the student characteristics, lower-extremity kinematics, and strength could be considered risk factors for a lower-extremity lesion in preprofessional contemporary dancers. The conclusions achieved for one year and multivariate analyses showed that in 82.2% of lower-extremity identified injuries, the ankle dorsiflexion was a risk factor for substantial lower-extremity injuries.

Regarding voice problems, several studies have been found. Devadas et al. [25] have researched the voice problems in India’s folk theatre artists, called Yakshagana. Singers reported voice problems in 91.2%. Actors reported only in 74%. Moreover, these problems provoked that artists missed work between 2 and 3 days of work. Results suggested that the Yakshagana artists should receive voice care education classes to prevent voice problems. Nehring et al. [28] investigated the exposure to classroom sound pressure levels among dance teachers. They found that the average Sound Pressure Levels (SPLs) in the dance classes were below or equal to the recommended limit; however, for some classes as tap or street dance, the SPLs reached values above 85 dB (A). Another study researched the hearing threshold of dance teachers [28]. Results showed a low prevalence of hearing loss for dance teachers, and also, the sex variable had affected the results only in the frequency of 9000 Hz. Other studies have proposed the need to integrate the schools and teachers to adapt the acoustical conditions to the needs of the students and the subjects [22,29,30], although these proposals have been mainly carried out in Italy. 

Based on the literature review results, the current study had as objective to determine the noise levels and thermal conditions existing in the workplaces, comparing to the adequate conditions following legal criteria, and the effect on dance teacher’s health from an ergonomic perspective. The main aim of this paper is to study the environmental work conditions for flamenco teachers in a case of study, correlating the results with the survey about safety and health of flamenco teacher in all Andalusian dance conservatories. A total of six dance conservatories have participated in this research achieving a total of 183 survey respondents.

## 2. Materials and Methods

### 2.1. Methodology

#### 2.1.1. Design and Sample

A cross-sectional study using a reference population of teachers from Andalusian conservatories was carried out. The samples were selected by a non-probabilistic procedure and classified by city. The recruitment was carried out in-person, by the authors, from March to June 2018.

The educational centers have different educational and pedagogical features, such as audio systems. There is no school health professional or a specifically designated health area within the educational centers. Nevertheless, the health center and general practitioner were close to the schools, and the town also has an emergency health professional.

The sample size calculation was made using the prevalence of health problems among dancers (around 45%) [6]. The Epidat 3.1 (Service of Information about Public Health (Xunta de Galicia, Spain)) [31] was used to carry out the sample calculation. Based on the sample of teachers in the conservatories from Andalusia, a sample size of 151 subjects randomly selected would suffice to estimate with a 95% confidence and a precision ± 5 percent units, a population percentage considered to be around 46%. The criteria used to determine the estimation was Andalusian dancer centers which would have similar conditions since these centers would be following the legal framework of the region. Finally, a sample of 175 teachers filled the survey. 

Additionally, the details about the structure, building, and operation of a conservatory of dance were considered to determine how the environmental conditions derived from the existence of noise, both in the center and inside the classrooms, the thermal conditions, and the exposure to vibration (due to repetitive movements) which take place due to heel taping, playing castanets, the piano, or the flamenco guitar affect the teaching staff. For such purpose, it was necessary to identify the different sources and types of noises in the conservatory, measuring the noise rates in those classrooms where backup instruments are used, as well as measuring the parameters that defines thermal environment following the international standards.

#### 2.1.2. Procedure

Participants approved a participant information statement, consent form, and survey. The first or initial page focused on the participant information statement and voluntary bases of the study, including the study’s objective, the explanation of the survey, the voluntary and consent to participate, and anonymity.

This informed consent followed the fundamental principles established in the Declaration of Helsinki of 1964, of the World Medical Association, subsequent amendments, and the 1996 Council of Europe Convention on Human Rights and Biomedicine and the Data Protection Law 3/2018 from the 5th of December. Moreover, this study was framed in an Occupational Safety project that received Ethical Research Approval (Reference 4258). Moreover, the authors received permission to gather the noise and thermal data to determine the working environment conditions’ relevance. 

#### 2.1.3. Statistical Analysis

After obtaining all the data, the Microsoft excel version 2018 (Microsoft Corporation, Redmond, WA, USA) and SPSS program version 25 (IBM Corporation, Armonk, NY, USA) were used to analyze such information.

The categorical variables were described by their absolute and relative frequency and the measures of central dispersion (mean, median, confidence intervals at 95% (CI), and interquartile range (IQR)). To compare the goodness-of-fit to an average distribution of data from continuous or discrete quantitative variables, the Shapiro–Wilk test and the Kolmogorov–Smirnov were applied accordingly to the variables, and the Levene test contrasted the homoscedasticity of variances. The normalization tests indicated that the data did not follow normality (*p* < 0.01). For the comparison of two independent arithmetic means, the Mann–Whitney U test was used, as indicated. The chi-square U, Kendall’s tau-b, Phi and Cramer’s V, and Spearmen’s correlations tests were used accordingly. Furthermore, multivariant models, such as regressions, were used accordingly. The Cronbach’s alpha test was used for determining the reliability of the survey, showing a really good reliability of the test to measure the workers’ perception (0.85).

#### 2.1.4. Analysis of Thermal Conditions 

Consideration of the consequences on the teacher health’ of an unsuitable thermal environment can be made from a hygienic or ergonomic point of view. The hygienic approach has already been exposed by applying the method contained in the ISO 7243:2017 standard.

The same type consequences from an ergonomic point of view should be performed considering the ISO 7730:2005 standard mentioned above. For that, the individual people model will be considered to comply with the cited standard, for the female gender, since there is a large majority of teachers who are women. This is: age 30 years old, body mass 60 kg, height 1.70 m, with a body surface area of 1.6 m^2^.

The metabolic rate should be considered as very high. No previous studies have been found that ratify this extreme for flamenco dance, but for similarity, in Pole Dance, Nicholas J. et al. [32] establishes 330 W. On the other hand, for pasodoble, or for samba and rumba, Zanchini and Malaguti [33] sets 490 W. This coincides, by approximation, with what is stated in Appendix A of the ISO 8996:2004 standard “Ergonomics of thermal environment. Determination of metabolic rate” [34], for similar activities, with metabolic rates of 520 W.

To reach this conclusion, a flamenco dance class has been modeled from the “Weekly curriculum of a flamenco dance class”, following the Order of 10-25-2007 [35] by which the curriculum of professional teachings of dance in Andalusia (Spain). For a 90-min class:

- Exercises for warming feet movements: 20 min;
- Exercises in the diagonal: practice of twists and pirouettes with stomping: 30 min;
- Work on the repertoire of flamenco dance: 40 min.

The teacher works all the elements continuously so that the students can see and learn. Of these three parts, we can highlight by metabolic load, the 40 min repertoire of flamenco dance, with high intensity.

### 2.2. Measurements, Equipment, and Instruments

#### 2.2.1. Sound Quality and Noise

The sound quality gives more details than the only sound level because this depends on how the human perceives it. One metric for quantifying how annoying is a sound is loudness. The loudness calculation entails the consideration of other aspects that depend on the power spectrum of a sound, this not being just sound pressure level, i.e., the critical bandwidth [36]. 

Zwicker Loudness gives equal-loudness contours with the same level describing the sensations of sound perceived by a person according to specific listening conditions used in this research. 

A multichannel equipment LMS SCADAS mobile and array of 36 ¼” microphones model TL-AHW 16.1, equipped with a digital camera, were used to measure noise emissions. The LMS SCADAS includes a 24 channels module, model SCM-V24, two eight channels modules, model SCM-V8-E, and one eight channels module, model SCM-VC8-E. The software used was LMS Test. Lab, by which it was calculated the Loudness Zwicker based on ISO 532 B for the diffuse field. Another parameter used to give information about the noise level was the overall level (SPL) in dB (A) in steady-state points. 

Measures were made in the classroom 03, 21 y 31 of the conservatory. The array of microphones was situated 2 m retired from any parament, and 2 m approximately from the dancers’ group, staying static during all measures.

#### 2.2.2. Thermal Conditions

For measuring thermal conditions, a WBGT (Wet Bulb Globe Temperature) monitor was used from Casella, model Microtherm. The information registered in every measure point was:

- T_a_: dry temperature;
- T_g_: globe temperature;
- T_nw_: natural wet temperature.

For measuring air velocity indoor the classroom, a meteorological station from TSI Instruments Ltd. (Shoreview, MN, USA), model VelociCalc 9545, was used.

Through the combination of these parameters, the WBGT index was obtained, following the ISO 7243:2017 “Ergonomics of the Thermal Environment—Assessment of heat stress using the WBGT index”. Being indoors, only T_g_ and T_nw_ were considered. These parameters also allow applying the PPD method described in the ISO 7730:2005 standard (Ergonomics of the thermal environment. Analytical determination and interpretation of thermal comfort using calculation of the PMV and PPD indices and local thermal comfort criteria) [37].

Measures was obtained putting the WBGT monitor on a tripod, in the center of each classroom, at 1.5 m above the floor (approximately torsos´ high). It has not been considered several measure highs because the verification of the vertical stratification temperature indicated the same temperature between floor and 2 m high, with difference inferior to 1 °C. Classrooms considered were numbers 3, 21, and 31, due that its situation into the building make it significative of all classrooms existing in the conservatory. Air velocity was measured putting the probe in several points around the classroom, for obtaining the higher air velocity indoor.

#### 2.2.3. Survey

A questionnaire was carried out to know how the conservatories’ perception of health and safety from the dance teachers’ and musicians’ points of view. The same questionnaire was given to the dance teacher, musicians, and singers of different conservatories of Andalusian (Spain) as Almeria, Cadiz, Cordoba, Granada, Malaga, and Seville. The questionnaire was divided into five sections (Appendix A) show the first page of the survey where the initial information is given to respond to the questionnaire as scales in the responses. Different kind of scales was used such as 6-point scale, ranging from 1 (respectively: 1—never, 2—sometimes, 3—usually, 4—almost always, and 5—always) to do not know/no answer; 3-point scale, ranging from 1 (respectively: 1—inappropriate, 2—acceptable, and 3—optimal) to 3. Moreover, the dichotomic variables with answers as no and yes were coded as 1 and 2, respectively, with the option of not knowing or missing as 0. In the beginning, the survey respondents were asked about their age, gender, educational level, and the type of teaching in the conservatory or if these are guitarists, pianists, or singers. 

Besides, the type of contract was also asked to match up the stable job or temporary with the injuries or illness. Regarding the health and safety measures, there were some questions about the knowledge related to prevention risk in the conservatory and about the noise and thermal conditions of the facilities, see Appendix A. Moreover, the dance teachers and staff were asked about their diseases, pathologies, and injuries due to their work and their careers to correlate them with the conservatories’ safety and hygienic environment. Finally, one free answer question about the surgical interventions that the teachers and musicians would have had, and the last question is a free answer question to write some commentaries considered important. 

## 3. Results

### 3.1. Noise Results

**Figure 2:**
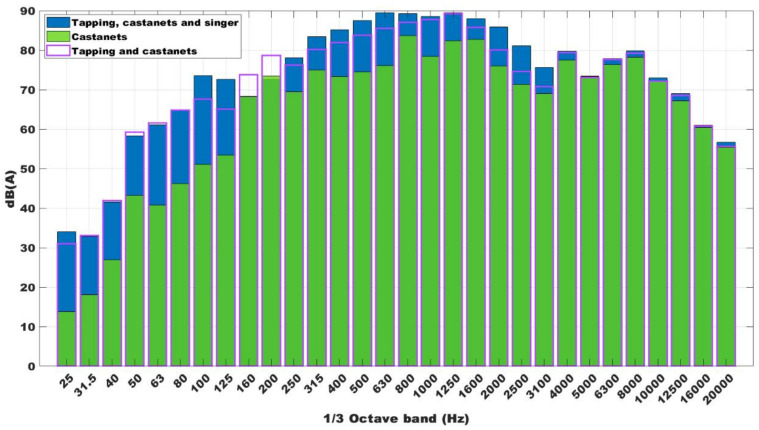
One third of octave spectrum for several combinations of noise sources: castanets, tapping, and singer (blue); castanets (green); and castanets and tapping (purple).

The measurements were carried out in a flamenco dance class with 14 students corresponding to the subject of bolero dance. The class duration was about one hour, using a special kind of floor to tap dance. Moreover, the music was live with a flamenco singer and guitarist. For this flamenco class, the students used castanets. Thus, the results were given for these three scenarios: castanets, tap heels, and singer and guitarist; only castanets; and castanets and tapping. Figure 2 shows the results for these three situations. In this figure, we can see that the use of castanets increases the high-frequency noise.

**Figure 3:**
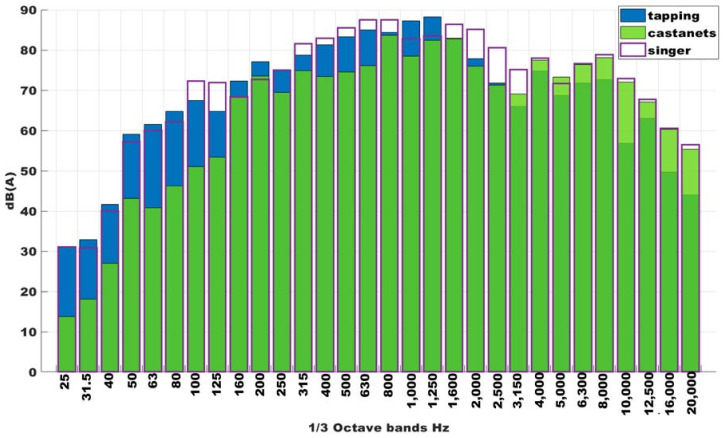
One third of octave spectrum for separated noise sources: tapping (blue); castanets (green); and singer (purple).

It should consider that measures realized for each combination of sound sources indicated before, are independent. That is, is not possible to compare exactly the values of each band in each sound source, but only the trends. Although the castanet player, or the dancer when tapping on the floor, try to make approximately the same sound, is impossible to be exact the same sound. Is for that, in Figure 3 we can observe that in some frequency bands, the sound of castanets and tapping, for example, is above the sound of castanets, tapping, and singer.

In its turn, the tapping increases the low-frequency noise. The highest noise level is achieved when castanets, tapping, singers, and guitarists were together, reaching above 90 dB(A) in the 800 Hz one third band, and up to 95 dB(A) in the global level. This is a high sound level during 5 h throughout the day, and it is the same for all days per week. Not in all classes there is live music, in some there is audio equipment that is used, and the volume of music is established by each teacher in their classes. Some authors have found that this could have consequences in hearing loss [26]. Following the results obtained in the survey explained in Section 3.3, almost 20% of the participants had temporary auditory threshold shifts at 500 Hz, 2000 Hz, and 6000 Hz. These results have been ratified in this research, following the survey results. Moreover, the maximum value was 97 dB(A), and the minimum value achieved during the recorder time was below 54 dB(A). Measures was made following the RD 286/2006 [38].

The Figure 3 shows the one third of octave spectrum for separated noise sources. It is possible to observe how the main noise source is the tapping, especially at low frequencies. Besides, the LAeq (equivalent continuous sound pressure level) reaches is 102 dB (A) considering all noise sources (tapping, castanets, and singer) which is higher than the established one by RD 286/2006 [38] (85 dB (A)). 

**Figure 4:**
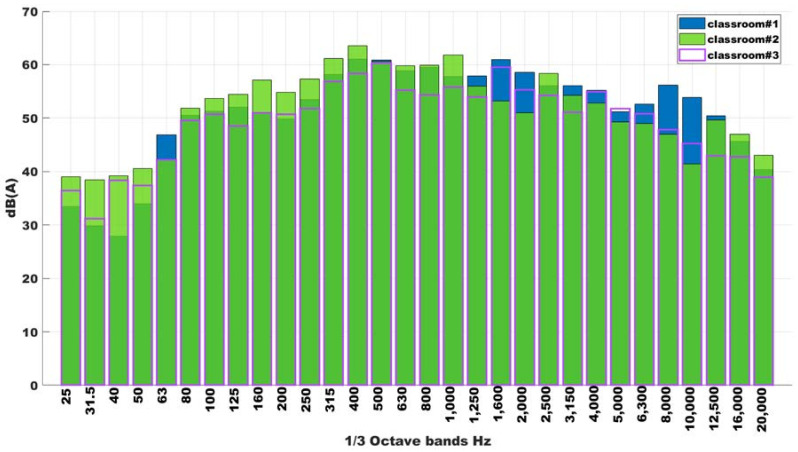
One third of octave spectrum of background noise for three different classrooms.

Figure 4 shows the background noise measured in three different classroom and comparing it with Figure 3, it is possible to see how the different is higher than 10 dB(A); thus, this could consider that the background noise is insignificant.

**Figure 5:**
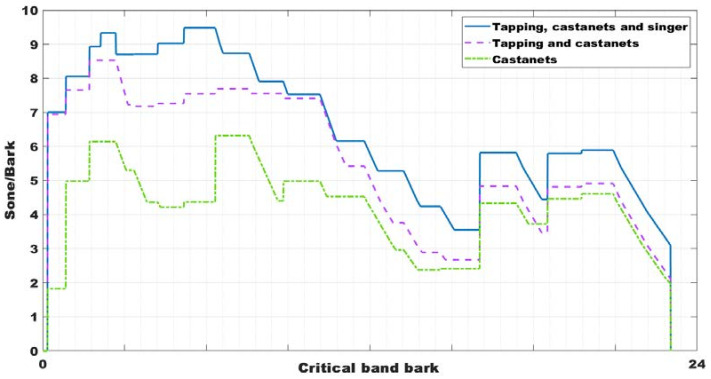
Specific Loudness of Zwicker in several noise sources combinations: castanets, tapping, and singer (blue); castanets (red); and castanets and tapping (green).

The specific loudness of Zwicker was also evaluated. As it is possible to observe in Figure 5, when castanets, tapping, singer, and guitarist were together, the maximum value was reached (around 9.20 sone/bark). Notably, castanets, tapping, and singer sound (blue outline) is predominated as specific loudness Zwicker on the others sound sources. 

### 3.2. Thermal Environmental Results

To consider the thermal environment, measurements were made during a full academic year, from November 2018 to July 2019. The measurements were carried out in three classrooms of the conservatory, the typical deviation of the measurements between classrooms being less than 1 °C. Monthly averages for natural wet temperature (T_nw_), globe temperature (T_g_) air velocity, and WBGT index, are in Table 1.

Choosing an average value of ambient temperature of those that can be considered more favorable from a thermal environment point of view, and a normal clothing in a flamenco dance class, we would consider:

- Operative Temperature: 16 °C;
- Thermal isolate for clothing: 0.5 clo;
- Air velocity in the classroom: below 0.1 m/s;
- Metabolism: Considering Table E.9 from the ISO 7730:2005 standard, with highest metabolism (232 W/m^2^) considering the standard people described before, it would be a metabolism of 371 W, even lower than that estimated for this type of dance and justified above. With all these data, following the calculation methodology of the ISO 7730: 2005 standard, a final PMV index of 1.27 has been obtained, which gives a PPD (percentage of people in discomfort) of around 40%. This result must be considered very high. This allows the conclusion that, from an ergonomic point of view, the thermal environment conditions are very demanding, although it is true that this is given by the very nature and metabolic rate of the activity that the teacher must develop during class.

From the hygienic point of view, and applying the ISO 7243:2017 standard, it is observed that in the months of June and July, the existing thermal environment levels are intolerable for periods of continuous activity of 40 min, which represents a clear worsening of the working conditions that these teachers must endure.

### 3.3. Surveys Results

**Figure 6:**
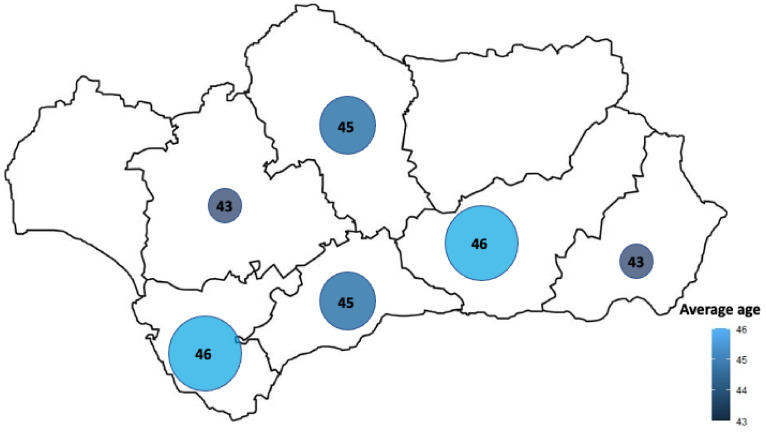
Distribution of dance teachers’ ages in the conservatories.

A total of 183 teachers responded to the survey. The city with a higher frequency of participants was Seville, with 20.7% (representing 38 teachers out of 183), followed by Granada (20.2%; 37 teachers out of 183), Cordoba (18.0%; 33 teachers out of 183), Malaga (15.3%; 28 teachers out of 183), Cadiz (14.2%; 26 teachers out of 183), and Almeria (11.5%; 21 teachers out of 183). The rate of response in Cordoba conservatory was 100% of the workers, while in Granada was 94.8% (being filled by 37 out 39), followed by Cadiz (74.3%; being 26 out of 35 teachers), Sevilla (around a 70% including professors and musicians answered the survey, being 38 out of 54), and, finally, Málaga and Almería which frequency was in 59.6% (being 28 out of 47) and 65.6% (21 teachers out of 32), respectively. The mean age of the survey respondents was around 45 years old, see Figure 6.

**Figure 7:**
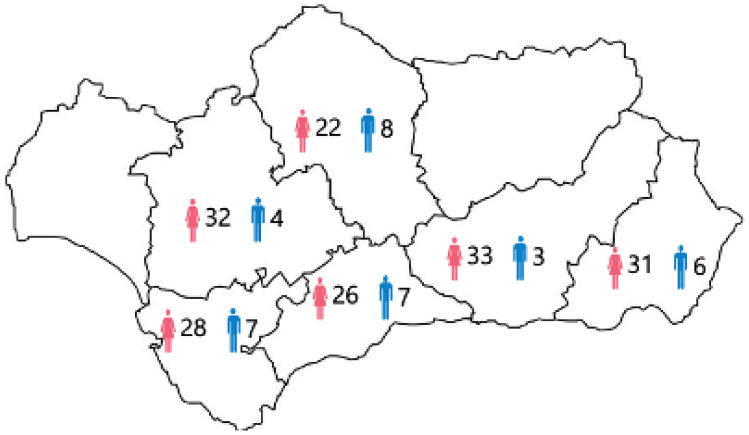
Gender of the dance teachers in the conservatories.

Figure 7 shows the gender of dance teachers in the conservatories. It is possible to appreciate that the number of men is lower than women, this percentage being a decrease of 20%, and having significant differences between samples (*p* < 0.05), although the sex was not linked to health issues (*p* < 0.05).

The results of each survey indicated differences between each city for the normative regarding occupational safety (*p* < 0.01), the self-preservation plan (*p* < 0.01), the risk associated with the work (*p* < 0.001), and the center’s update of the occupational safety and health protocols (*p* < 0.001). There were not found differences between centers regarding the evaluation given to the working environment, the presence of health issues (injuries or discomfort and problems associated with noise), annual medical check-ups, or the type of contract (*p* > 0.05).

Besides, the presence of health problems or issues was found to be presented in 84.6% of the sample, being the mean of health problems set in 6.3 ± 4.6 95% CI (5.6–6.9). Moreover, the presence of health issues seemed was different in multiples variables according to the chi-test (Table 2). The frequency of the factors showed how most participants had an indefinite contract (70.3%), considered that they knew the normative regarding occupational safety (45.1%), the self-protective code of the center (57.7%), and the risks associated with the work (73.1%). Moreover, the correlations indicated a link between having a health issue and having an indefinite contract (ρ = 0.657; *p* < 0.001), not knowing the normative (ρ = 0.387; *p* < 0.001), the self-protection plan (ρ = 0.356; *p* < 0.001), and not knowing the risk associated to work (ρ = 0.465; *p* < 0.001).

The participants’ health issues were also linked to their opinion regarding the prevention measures taken by the centers (Table 3). Table 3 showed how the perception of workers with health issues about the occupational safety and health (OSH) measures taken by the center (median = 2; IQR = 1), the training regarding OSH measures (median = 2; IQR = 2), the actualization (median = 2; IQR = 4), or medical check-ups (median = 2; IQR = 2.75) were never or sometimes carried out in the working environment. Table 3 also indicated how there were significant differences between perceptions among the workers. Moreover, the correlations indicated how workers with health issues had a tendency to indicated that sometimes the OSH measures were applied (τ = −0.31; *p* < 0.001), the training regarding OSH in working hours (τ = −0.41; *p* < 0.001), the additional information (τ = −0.29; *p* < 0.001), the actualization of the protocols (τ = −0. 344; *p* < 0.001), and the annual medical check-ups (τ = −0.29; *p* < 0.001).

The participants’ health issues were ultimately associated with their opinion regarding the evaluation of the medical check-ups and the working conditions, focusing on noise, thermal environment, and vibrations (Table 4). The evaluation of workers with health issues regarding the medical check-ups and environmental conditions was unacceptable (median = 1; IQR = 1), although there were no significant differences between those participants with better opinion and worse opinion regarding the medical check-ups (Table 4). The correlations indicated how workers, that considered they had no health issues, had a better opinion about the climatologic (ρ = 0.55; *p* < 0.001) and thermal (ρ = 0.55; *p* < 0.001) conditions, the air stream in classrooms (ρ = 0.43; *p* < 0.001), the humidity (ρ = 0.2; *p* < 0.05), the acoustic for communicating with the students (ρ = 0.39; *p* < 0.001), noise levels (ρ = 0.43; *p* < 0.001), and acoustic isolation in the classrooms (ρ = 0.34; *p* < 0.001).

A multivariant analysis (R square = 0.56; sum of squares = 8.7; *p*-value < 0.001) showed that the presence of any health issue in this samples was linked to having an indefinite contract (*p* < 0.001), knowing the normative regarding occupational safety and health (*p* < 0.001), and the risk associated to work (*p* < 0.01), being the training about OSH scheduled during the working hours (*p* < 0.05), and the annual medical check-ups (*p* < 0.001).

The health issues were divided into injuries (being presented in 89.7%) and discomfort and problems associated with noise (being presented in 68.0%), being linked between each other (ρ = 0.45; *p* < 0.001). The mean of motion injuries was higher (3.88 ± 2.7) per worker than the discomfort and problems linked to noise levels (2.7 ± 2.5). Table 5 showed the injuries associated with ergonomic and repetitive motion during the dance classes. The injuries with the most incidences for all conservatories in Andalusia was muscle contracture. Moreover, cramps had a high incidence rate as injury among dance teachers and musicians. On the contrary, the fractures were the least frequent injuries for dance teachers. The conservatories with the highest rate of lesions were Cordoba and Granada. However, the percentages in Cadiz and Almeria showed the lowest rate of repetitive motion injuries. Additionally, no significant differences were found between injuries associated with the ergonomic and repetitive motion during the dance classes and the different cities (*p* > 0.05).

The injuries associated to the ergonomic and repetitive motion was linked to the evaluation of the climatologic (ρ = 0.49; *p* < 0.001), thermal conditions (ρ = 0.52; *p* < 0.001), air stream (ρ = 0.42; *p* < 0.001), humidity (ρ = 0.23; *p* < 0.01), acoustic for communicating students (ρ = 0.38; *p* < 0.001), noise levels (ρ = 0.41; *p* < 0.001), and the isolation (ρ = 0.38; *p* < 0.001). Moreover, the injuries were also associated to having an indefinite contract (ρ = 0.52; *p* < 0.001), the normative of OSH in the center (ρ = 0.31; *p* < 0.001), the self-protection plan of the center (ρ = 0.21; *p* < 0.01) knowing the risks related to the work (ρ = 0.37; *p* < 0.001), and the frequency of training, additional information, or actualizations regarding OSH and medical check-ups (*p* < 0.001). The multivariant analysis (R square = 0.54; *p*-value < 0.001; with a constant of B = 0.81) showed that the presence of lesions or ergonomic problems due to motion was associated to having an indefinite contract (*p* < 0.001), knowing the risk associated to the work (*p* < 0.01), being the training about OSH scheduled during the working hours (*p* < 0.01), the actualization of protocols *(p* < 0.001), the annual medical check-ups (*p* < 0.001), the evaluation of air stream (*p* < 0.001), the acoustic for communicating with students (*p* < 0.001), and the noise levels in the classrooms (*p* < 0.001).

Regarding the discomfort and problems associated with noise during the classes, Table 6 showed the annoyances for Andalusian’s different conservatories. The main problem associated with high noise is discomfort, vocal nodules, and edema or swellings. Deafness, irritability, and stress problems were also presented for all conservatories except Cadiz, where the percentage for these is slightly lower. However, sleep disturbances seem not to be a problem among the dance teachers, musicians, and singers as this has an average percentage of around 20%. Although, significant differences were found between its presence and the different cities (*p* > 0.05).

The discomfort and problems associated to noise was linked to the evaluation of the climatologic (ρ = 0.64; *p* < 0.001), thermal conditions (ρ = 0.64; *p* < 0.001), air stream (ρ = 0.57; *p* < 0.001), humidity (ρ = 0.41; *p* <0.001), acoustic for communicating students (ρ = 0.50; *p* < 0.001), noise levels (ρ = 0.32; *p* < 0.001), and the isolation (ρ = 0.23; *p* < 0.01). Moreover, the discomfort and other health problems were also associated to having an indefinite contract (ρ = 0.73; *p* < 0.001), the normative of OSH in the center (ρ = 0.62; *p* < 0.001), the self-protection plan of the center (ρ = 0.17; *p* < 0.05) knowing the risks related to the work (ρ = 0.48; *p* < 0.001), and the frequency of additional information and actualizations regarding OSH (*p* < 0.001). The medical check-ups, the raining provided by the center and being such training schedule in the working hours were not linked to the presence of discomfort or health problems associated to noise (*p* > 0.001). The multivariant analysis (R square = 0.69; *p*-value < 0.001) showed that the presence of the discomfort or problems related to the noise were linked to having an indefinite contract (*p* < 0.001), having additional information regarding OSH in the centers *(p* < 0.05), the actualization of protocol regarding OSH (*p* > 0.01), the annual medical check-ups (*p* < 0.001), the evaluation of thermal conditions (*p* < 0.001), and the noise levels in the classrooms (*p* < 0.001).

## 4. Discussion

One of the main problems for dance teachers is the constant exposure to high sound pressure levels which may cause suffer hearing damage due to this long-term exposure [39,40]. For sound, pressure levels ranged between 85–90 dB(A) could have irreversible damage to the inner ear. These consequences depending on exposure time, being stablished by legal criteria that it would be hearing issues from 80 dB(A) of personal exposure, time-weighted per 8 h/day, five days per week; thus, this should be reduced using, for example, techniques of reorganization at work, or the amplitude of sound could also be reduced with acoustic insulation on the walls reducing the echo [39,40]. In this sense, the survey results indicated how the health issues, which were highly common in the teachers, were linked to noise exposure and thermal conditions, especially for discomfort or problems related to noise.

The RD 286/2006 [38] establishes a limit that should not be exceeded as a noise equivalent continuous level (L_Aeq_) of 85 dB(A) or a peak noise level of 137 dB(C), taking measurements as wear hearing protection. In this case, for dance teachers, the use of this kind of preventive measure could maybe be challenging to apply to both teachers and singers or musicians. The fact of that dance teacher and other staffs working in the conservatory could be exposed to a high level of noise in addition to those that may be exposed in their daily life increases the risk for suffering hearing damage [41], which is confirmed by the prevalence of the health issues linked to noise levels, such as deafness.

Around 54.50% showed general discomfort, a little more than 50% (51.53%) answered that they used to have a headache, and with a lower percentage (46.86%), they showed irritability or stress. Figure 3 showed that the noise registered during the dance classes reached up 90 dB(A), which was above the limit established [38]. These were some problems found in the conservatories, which could be due to the high SPL for a long time. However, the mean percentage of responses about the sleep disturbances was very inferior (20.23%), so this kind of problem could be found when people are exposed to night noise [27]. Another factor of risk associated with the classes’ high noise is the problem with the voice nodules. This is the only disease listed in the Ministry table [17] as an occupational disease. Over an average percentage of 49.97% of dance teachers and singers reported problems with vocal nodules or oedemas. All these health issues linked to noise were adjusted associated with evaluating thermal conditions and the noise levels in the classrooms.

Another critical factor to considerer is the average daily teaching load, which means it is around five hours per day [42]. This involves that dance teachers are exposed to high noise levels (>85 dB(A)) during long periods. It has been proved that continuous exposure to high SPL has adverse health effects and hearing impairment, such as disturb sleep, cause cardiovascular and psychophysiological effects, reduce performance, and provoke annoyance responses and changes in social behavior [39,40,41,43]. The previous literature seemed to match the current results from this study, highlighting how environmental conditions indices the health issues and the prevention measures, the occupational safety and health protocols, and medical checks-up were significantly connected to the health problems.

Hence, both dance teachers, singers, and musicians were informed about the importance of reducing the SPL and the risk for their health. Besides, each conservatory’s principal was reported about the benefits of isolating the classes to achieve a reduction of reverberation so reducing the SPL. Another recommendation given to the conservatories was the use of another kind of floor. The noise of metal hitting against floor could be different depending on the kind of floor, and it could also influence the injured suffer by the dance teachers and students. Malliou et al. [44] studied the dance aerobic instructors’ injuries concerning external risk factors. The authors concluded that the most important external factors were the excessive working hours, the high intensity of the classes, the style of dance, the elastic floor, and the deficient shoes.

Thermal environment conditions in the classrooms are also an important disturbing factor in the working conditions of teachers. As shown in Table 1, these conditions produce a very important stress. Applying the standard with hygienic criteria (ISO 7243:2017) [45], the thermal conditions in summer months are unacceptable, potentially putting the health of teachers at risk. On the other hand, applying the ergonomic criterion (ISO 7730:2005) [37], and applying the results obtained on the typical woman that defines the aforementioned standard, the result obtained is clearly negative from this perspective. It can therefore be affirmed that thermal environment in classrooms, with respect to the work that the teacher must develop, is clearly aggressive from both hygienic and ergonomic perspectives.

In this sense, the results from teachers indicated how the hygienic and ergonomic factors in the working environment, as well as knowing the OSH measures or the medical checkups, played a key role in their perception of health issues and injuries. The health issues and injuries were greatly common among participants, which initially seemed to be considerable difference between the injuries for each conservatory, but that indeed there were not significant between regions. Moreover, these results regarding the frequencies of injuries could be due to the kind of floor used or if the conservatory had been renovated its facilities recently. Van Seters et al. researched the risk factor for lower-extremity injuries among contemporary dance students [23]. They observed that there were differences between the injuries for bachelor dance teachers and bachelor dance students. It could be because of the differences in the education program where dance teacher students might be exposed to a great deal of effort and less time recovering. Thus, proposing a new timetable with more rest between dance teachers was also a recommendation given to the conservatories to prevent our study’s injuries.

This study’s sample included whole Andalusian dance conservatories was mainly female participants; although this is the reality of most dance conservatories where both female dance teachers and students prevail. In this sense, as previous studies have found [28], men’s dance teachers are a minority, this being an important issue. Regarding the age, the average age was over 40 years. It could be considered that this average age is much higher compared to other studies where this is around 30 years [46]. Moreover, this fact goes against the theory that many of these professionals have to abandon dance because they grow older but become active on a different level. In Spain, the average age of dance teachers to retire could be until 70 years, although the reality is that most of them retire fifty.

Like any study, there are limitations. The survey data is based on people’s opinions with a transversal cut and should be wise to apply the results without contemplating the differences between samples. Additionally, because of the state and content of the survey, the participants were not asked how exposed to the factors. Therefore, some associations could be further or less associated with possible bias factors in the models.

Despite the limitations, the current study presents an innovative perspective combining different measurements and a specific survey and being applied for a population with little-studied environmental factors. This approach was not being made previously since more studies focused on establishing health problems and noise measures but did not include the perception in different moments, including individual’s, building’s factors, and possible originators of noise.

## 5. Conclusions

In this study, a survey was carried out in the Andalusia dance conservatories, and noise, thermal conditions, and characteristics of buildings are considered, as influence factors on health of teachers. Moreover, a survey was carried out in all the high professional conservatories in Andalusia (Spain) between the teachers, asking about injuries and other effects of their working conditions.

The noise was measured for different kinds of flamenco dance classes. The results have corroborated that the high sound level and lack of measures for occupational safety and health have consequences on dance teachers, musicians, and singers’ health. The sound levels achieved were higher than 85 dB (A), and some measurements were proposed. Furthermore, the higher Zwicker Loudness values were found at low-mid frequencies. The reduction of the hours of classes, an increase in test time turns down the music volume when it could be possible. Besides, the renovation of the floor for other more appropriate with more damping has been recommended.

Thermal conditions were supervised for a whole academic year. We can conclude that the conditions are no adequate, especially in summer, considering the existent temperatures in Córdoba (Spain) and the deficient isolation from outdoors in the buildings, from hygienic and ergonomic international standards point of view.

The results of the survey had showed a relationship between the kind of contract, not knowing the normative or prevention risks in their conservatories and health problems. Other key factor was that the participants’ health issues were ultimately associated with their opinion regarding the evaluation of the medical check-ups and the working conditions, focusing on noise, thermal environment, and vibrations.

All that factors affect to the health of teachers, as is reflected in the statistical analyses realized, with a high statistical significance level.

## Figures and Tables

**Table 1:** Monthly averages for natural wet temperature (T_nw_), globe temperature (T_g_), air velocity (Var), and WBGT (Wet Bulb Globe Thermometer) index.

Month	Parameters	Classroom 03	Classroom 21	Clasroom 31
November	Tg (°C)	16.4	16.1	15.6
	Tnw (°C)	16.2	15.7	15.2
	WBGT (°C WBGT)	16.3	15.8	15.3
	Var (m/s)	<0.1	<0.1	<0.1
December	Tg (°C)	15.5	16.7	15.2
	Tnw (°C)	14.8	14.6	13.6
	WBGT (°C WBGT)	15.0	15.2	14.1
	Var (m/s)	<0.1	<0.1	<0.1
January	Tg (°C)	14.5	13.4	14.3
	Tnw (°C)	13.5	12.9	13.4
	WBGT (°C WBGT)	13.8	13.0	13.7
	Var (m/s)	<0.1	<0.1	<0.1
February	Tg (°C)	15.2	14.8	14.7
	Tnw (°C)	13.6	14.6	14.7
	WBGT (°C WBGT)	14.1	14.6	14.7
	Var (m/s)	<0.1	<0.1	<0.1
March	Tg (°C)	16.7	17.3	17.6
	Tnw (°C)	16.3	16.5	16.6
	WBGT (°C WBGT)	16.4	16.7	16.9
	Var (m/s)	<0.1	<0.1	<0.1
April	Tg (°C)	23.6	23.2	24.0
	Tnw (°C)	21.3	21.0	23.0
	WBGT (°C WBGT)	21.9	21.6	23.3
	Var (m/s)	<0.1	<0.1	<0.1
May	Tg (°C)	23.3	23.7	24.9
	Tnw (°C)	20.1	20.6	24.8
	WBGT (°C WBGT)	21.0	21.5	24.8
	Var (m/s)	<0.1	<0.1	<0.1
June	Tg (°C)	30.5	30.0	28.2
	Tnw (°C)	27.5	26.9	28.2
	WBGT (°C WBGT)	28.4	27.8	28.2
	Var (m/s)	<0.1	<0.1	<0.1
July	Tg (°C)	30.8	30.2	29.1
	Tnw (°C)	27.9	27.4	28.9
	WBGT (°C WBGT)	28.8	28.2	29.0
	Var (m/s)	<0.1	<0.1	<0.1

**Table 2:** Perception of health issues in the teachers and its association to the presence or not of other factors.

Health Issues	Yes	No	*p*-Value
Indefinite contract	123 (83.1%)	25 (16.9%)	<0.001
Knowing the normative regarding occupational safety and health	79 (53.4%)	69 (46.62%)	<0.001
Self-preservation plan	96 (64.8%)	69 (35.2%)	<0.001
Risk related to the work	121 (81.8%)	27 (18.2%)	<0.001

**Table 3:** Perception of health issues of the teachers (N = 149) and its association to occupational and health factors regulated by centers.

Factors	Rather Not Say	Never	Sometimes	Usually	Almost Always	Always	*p*-Value
Frequency that the OSH (Occupational Safety and Health) are applied in the center	5 (3.3%)	47 (31.5%)	57 (38.3%)	16 (10.7%)	6 (4.0%)	18 (12.1%)	<0.001
The training of OSH measures is scheduled during the working hours	7 (4.7%)	43 (28.9%)	55 (36.9%)	13 (8.7%)	5 (3.3%)	19 (12.6%)	<0.001
The center receives additional OSH information	50 (33.6%)	33 (22.1%)	37 (24.8%)	17 (11.4%)	5 (3.3%)	6 (4.0%)	<0.001
The center actualizes the OSH protocols and normative	51 (34.2%)	15 (10.1%)	24 (16.1%)	12 (8.1%)	18 (0.1%)	28 (18.8%)	<0.001
The medical check-ups are annually carried out	14 (9.4%)	52 (34.9%)	28 (18.8%)	14 (9.4%)	13 (8.7%)	27 (18.1%)	<0.001

**Table 4:** Perception of health issues in the teachers and its association to the opinion for the working environment.

Factors	Do Not Answer	Unacceptable	Acceptable	Optimal	*p*-Value
Medical check-ups	20 (10.3%)	78 (52.3.3%)	45 (30.2%)	6 (4.0%)	>0.05
Climatologic conditions	1 (0.7%)	124 (83.2%)	24 (28.9%)	0 (0%)	<0.001
Thermal conditions	1 (0.7%)	131 (87.9%)	17 (22.1%)	0 (0%)	<0.05
Airstream	1 (0.7%)	107 (71.8%)	39 (22.1%)	2 (1.3%)	<0.001
Humidity	16 (10.7%)	78 (52.3%)	50 (10.1%)	5 (3.4%)	<0.001
Acoustic for communication with students	1 (0.7%)	91 (61.1%)	50 (33.6%)	7 (4.7%)	<0.001
Noise levels	1 (0.7%)	122 (81.9%)	19 (12.7%)	7 (4.7%)	<0.001
Acoustic isolation in the classrooms	1 (0.7%)	117 (78.5%)	20 (13.4%)	11 (7.4%)	<0.001

**Table 5:** Repetitive motion injuries in the Andalusia conservatories.

Repetitive Motion Injuries	Total Prevalence	Seville	Malaga	Cordoba	Cadiz	Granada	Almeria
Tendinitis	84.60%	86.11%	84.62%	83.33%	76.67%	86.67%	46.67%
Fracture	12.60%	8.33%	7.69%	10.00%	3.33%	33.33%	10.00%
Muscle tear	40.60%	40.54%	26.92%	40.00%	33.33%	63.33%	26.67%
Herniated discs or protrusions	37.70%	21.62%	23.08%	63.33%	20.00%	53.33%	26.67%
Deformations (feet, fingers)	37.70%	37.84%	26.92%	33.33%	33.33%	60.00%	23.33%
Ligament strains	37.10%	35.14%	34.62%	26.67%	30.00%	60.00%	26.67%
Cramps	49.70%	45.95%	42.31%	43.33%	33.33%	76.67%	43.33%
Muscle contractures	88.60%	86.49%	84.62%	83.33%	73.33%	100.00%	56.67%

**Table 6:** Discomfort and problems associated with noise in the classes.

Discomfort	Total Prevalence	Seville	Malaga	Cordoba	Cadiz	Granada	Almeria
General discomfort	58.90%	51.35%	42.31%	66.67%	83.33%	46.67%	36.67%
Headaches	55.40%	48.65%	53.85%	66.67%	36.67%	63.33%	40.00%
Irritability. stress	51.40%	62.16%	42.31%	60.00%	20.00%	53.33%	43.33%
Sleep disturbances	21.10%	21.62%	23.08%	6.67%	16.67%	33.33%	20.00%
Deafness	36.00%	40.54%	38.46%	40.00%	16.67%	50.00%	26.67%
Vocal nodules, or edema or swellings	52.00%	45.95%	53.85%	53.33%	43.33%	70.00%	33.33%
Others	2.30%	0.00%	0.00%	0.00%	0.00%	0.00%	13.33%

## Data Availability

The data is available for researchers, please contact the authors.

## Appendix A

**Sex:**

Men		Female		Age	

**Titulación académica** (sólo marque el máximo nivel alcanzado):

General

High school	
Certified	
Licensed/Graduated	
Postgraduate	
PhD	
Others	

Specify

	Superior	Professional
Classical dance		
Spanish dance		
Contemporary dance		
Flamenco		
Accompanying guitarist		
Accompanying singer		
Accompanying pianist		
Other/s		

**Current position**

Interim substitute or vacant	
Civil servant trainee	
Vacancy officer	
Definite officer	

**Teaching experience**

Levels that gives lessons:

Basic Education	
Professional Teachings	
Both	

Specialty for which you are attached to the center

Subjects taught

	Yes	No	
I know the applicable regulations on the prevention of occupational hazards.			
I know the Center’s Self-Protection Plan.			
I know the professional risks associated with the work I do.			

	1 Never	2 Sometimes	3 Usually	4 almost always	5 Always	0 Rather not say/Not known
Teachers request training from the center about Occupational Risk Prevention and safety, and health.						
The center’s programs specific training on prevention at affordable times.						
The center receives additional instructions to the regulations by different means on Risk Prevention.						

	1 Never	2 Sometimes	3 Usually	4 almost always	5 Always	0 Rather not say/Not known
The center updates and implements the measures contemplated in the Self-Protection Plan						
The teaching staff annually carry out the medical examination provided for in the Law.						

	1 Inadequate	2 Adequate	3 Optimo
How is the medical examination carried out by the Center for the Prevention of Occupational Risks considered for conservatories’ professionals?			

Thermal environment

	1 Inadequate	2 Adequate	3 Optimo
Please rate the weather conditions you find in the classroom for the work being done.			
Rate the classroom temperature conditions (hot-cold)			
Rate the draft and ventilation conditions in classrooms			
Rate the humidity conditions in the classroom.			

Noise levels

	1 Inadequate	2 Adequate	3 Optimo
Rate acoustic when communicating with your students.			
Rate the noise in the classroom due to the music, the accompaniment, the castanets, the tapping, etc.			
Rate the acoustic isolation of a classroom or facility concerning the noise generated in another.			

By repetitive movements and by the use of the accompanying musical instruments:

Tendinitis	
Fracture	
Muscle tear	
Herniated discs or protrusions	
Deformations (feet, fingers.…)	
Ligament strains	
Cramps	
Muscle contractures	

Due to the noise generated by the accompanying instruments and the work of shoes and castanets:

General discomfort	
Headaches	
Irritability. stress	
Sleep disturbances	
Deafness	
Vocal nodules, or edema or swellings	
Others	

Indicate if you have undergone any surgical intervention as a result of the work you do

Thanks for your input.
